# *Rahnella* sp., a Dominant Symbiont of the Core Gut Bacteriome of *Dendroctonus* Species, Has Metabolic Capacity to Degrade Xylan by Bifunctional Xylanase-Ferulic Acid Esterase

**DOI:** 10.3389/fmicb.2022.911269

**Published:** 2022-05-31

**Authors:** Rosa María Pineda-Mendoza, Gerardo Zúñiga, María Fernanda López, María Eugenia Hidalgo-Lara, Alejandro Santiago-Hernández, Azucena López-López, Flor N. Rivera Orduña, Claudia Cano-Ramírez

**Affiliations:** ^1^Laboratorio de Variación Biológica y Evolución, Departamento de Zoología, Escuela Nacional de Ciencias Biológicas, Instituto Politécnico Nacional, Mexico City, Mexico; ^2^Laboratorio de Ingeniería de Proteínas, Centro de Investigación y de Estudios Avanzados del Instituto Politécnico Nacional (CINVESTAV-IPN), Departamento de Biotecnología y Bioingeniería, Mexico City, Mexico; ^3^Laboratorio de Ecología Microbiana, Departamento de Microbiología, Escuela Nacional de Ciencias Biológicas, Instituto Politécnico Nacional, Mexico City, Mexico

**Keywords:** symbiosis, bark beetle, carbohydrates metabolism, CBM48, glycoside hydrolase, xylan hydrolysis

## Abstract

*Rahnella* sp. ChDrAdgB13 is a dominant member of the gut bacterial core of species of the genus *Dendroctonus*, which is one of the most destructive pine forest bark beetles. The objectives of this study were identified in *Rahnella* sp. ChDrAdgB13 genome the glycosyl hydrolase families involved in carbohydrate metabolism and specifically, the genes that participate in xylan hydrolysis, to determine the functionality of a putative *endo*-1,4-β-D-xylanase, which results to be bifunctional xylanase–ferulic acid esterase called R13 Fae and characterize it biochemically. The carbohydrate-active enzyme prediction revealed 25 glycoside hydrolases, 20 glycosyl transferases, carbohydrate esterases, two auxiliary activities, one polysaccharide lyase, and one carbohydrate-binding module (CBM). The R13 Fae predicted showed high identity to the putative esterases and glycosyl hydrolases from *Rahnella* species and some members of the Yersiniaceae family. The *r13 fae* gene encodes 393 amino acids (43.5 kDa), containing a signal peptide, esterase catalytic domain, and CBM48. The R13 Fae modeling showed a higher binding affinity to ferulic acid, α-naphthyl acetate, and arabinoxylan, and a low affinity to starch. The R13 Fae recombinant protein showed activity on α-naphthyl acetate and xylan, but not on starch. This enzyme showed mesophilic characteristics, displaying its optimal activity at pH 6.0 and 25°C. The enzyme was stable at pH from 4.5 to 9.0, retaining nearly 66–71% of its original activity. The half-life of the enzyme was 23 days at 25°C. The enzyme was stable in the presence of metallic ions, except for Hg^2+^. The products of R13 Fae mediated hydrolysis of beechwood xylan were xylobiose and xylose, manifesting an *exo*-activity. The results suggest that *Rahnella* sp. ChDrAdgB13 hydrolyze xylan and its products could be assimilated by its host and other gut microbes as a nutritional source, demonstrating their functional role in the bacterial-insect interaction contributing to their fitness, development, and survival.

## Introduction

Symbiotic associations between insects and microorganisms are ubiquitous in nature. Depending on the type of microorganism and biotic and abiotic factors, these associations can vary from mutualism to antagonism, and from obligate to facultative (Ferrari and Vavre, 2011). It is widely recognized that these symbioses have enhanced the capabilities of insects to exploit a plethora of resources, explore and colonize new habitats, and define new ecological niches. Symbiotic gut bacteria provide vital benefits to their hosts, such as nutrient supplementation, resistance to pathogens, altering the host’s reproductive system, assistance in the immunity modulation, and regulation of the microbial community (Kashkouli et al., 2021).

These symbionts can be acquired from the environment or transmitted by inheritance (Wein et al., 2019; Leftwich et al., 2020). The gut bacteria of *Dendroctonus*-bark beetles are environmentally acquired through food because these insects carry out their life cycle almost entirely under the bark of pine trees (Family Pinaceae) where they breed in and feed on the phloem tissue, a substrate rich in cellulose, hemicellulose, pectin, and lignin (Six and Bracewell, 2015; Gonzalez-Escobedo et al., 2018). These structural polysaccharides are complex molecules, whose hydrolysis involves both primary enzymes (e.g., endoglucanases and endoxylanases) and accessory enzymes (e.g., cellobiohydrolases, β-glucosidase, β-D-xylosidase, α-L-arabinofuranosidases, and ferulic acid esterase) (Walia et al., 2017; Chen et al., 2020).

In particular, xylan is a component of hemicellulose, which is the second polysaccharide in abundance in the cell walls of plants and some green and red algae (Scheller and Ulvskov, 2010; Hsieh and Harris, 2019). It is formed by the homopolymeric backbone chain of 1,4-linked β-D-xylopyranosyl units, which can be substituted by side-chain groups, such as glucuronic pyranosyl, 4-*O*-methyl-D-glucurono pyranosyl, α-L-arabinofuranosyl, acetyl, feruloyl, and/or *p*-coumaroyl (Walia et al., 2017; Bhardwaj et al., 2019). The breakdown of xylan and arabinoxylan are carried out by different enzymes: the *endo*-1,4-β-D-xylanase (EC 3.2.1.8) that randomly cleaves the xylan backbone, β-D-xylosidase (EC 3.2.1.37) that cleaves xylose monomers, whereas the removal of the side groups is catalyzed by α-L-arabinofuranosidases (EC 3.2.1.55), α-D-glucuronidases (EC 3.2.1.139), acetylxylan esterase (EC 3.2.1.72), ferulic acid esterase (EC 3.1.1.73), and *p*-coumaric acid esterase (EC 3.1.1.B10) (Walia et al., 2017; Bhardwaj et al., 2019; Chen et al., 2020; Houfani et al., 2020).

The xylanolytic activity of gut-associated microorganisms of insects has been reported in termites (Blattodea: Termitidae), wood borer (Coleoptera: Cerambycidae), and muga silkworm (Lepidoptera: Saturniidae) (Delalibera et al., 2005; Anand et al., 2010; Gandotra et al., 2018; Jang and Kikuchi, 2020). Given that there is no evidence of genes encoding for enzymes involved in xylan depolymerization in the genome of *Dendroctonus ponderosae*, nor in transcriptomes of several species of *Dendroctonus* (Torres-Banda pers. comm.), it is possible that this activity could be carried out by gut-associated microbes from bark beetles. In fact, recently, in the genome of two strains of *Rahnella* sp. isolated from bark beetle *Ips typographus* larvae were identified, which were able to degrade xylan in plates, and genes involved in xylan hydrolysis were identified (Fabryová et al., 2018).

The genus *Rahnella* (Yersiniaceae), which includes thirteen species isolated from diverse environments (e.g., water, seed, food, insects, plants, and clinical samples) (Brady et al., 2022), apparently is a symbiont critically important to *Dendroctonus* species, since plays a crucial role in nutritional and detoxification processes degrading substrates such as esters and lipids (Briones-Roblero et al., 2017), recycling uric acid (Morales-Jiménez et al., 2013) and transforming different monoterpenes present in the host trees of these insects (Boone et al., 2013; Xu et al., 2015, 2016). In addition, *Rahnella* is a dominant a persistent member of the core gut bacteriome of *Dendroctonus* bark beetles, along with genera *Pseudomonas, Serratia, Raoultella, Pantoea*, and *Enterobacter* (Hernández-García et al., 2017), which surely provide a series of benefits to these insects that increase their fitness (Six, 2013).

Therefore, the objectives of this study were identified in the *Rahnella* sp. ChDrAdgB13 genome, a strain isolated from the *D. rhizophagus* gut, members of the glycosyl hydrolase (GH) families involved in carbohydrate metabolism, especially genes that participate in the degradation of xylan. Based on these results, we characterize *in silico* the gene that codes to *endo*-1,4-β-D-xylanase, which results to be a gene from a bifunctional xylanase-ferulic acid esterase called *r13 fae*. Lastly, we determine the functionality of this xylanase-ferulic acid esterase and characterized it biochemically. This information will enable us to comprehend how *Rahnella* sp. ChDrAdgB13 and this enzyme participate in the breakdown of xylan.

## Materials and Methods

### Genome Analysis of Strain ChDrAdgB13

*Rahnella* sp. ChDrAdgB13 (accession number CDBB B-2057 = NRRL B-65604) was isolated from the emerged-adults gut of *Dendroctonus rhizophagus* infesting *Pinus arizonica* Engelm at San Juanito locality (27° 55′ 54.9″ N and 107° 35′ 54.6″ W; 2452 masl), Bocoyna Municipality, Chihuahua, Mexico (Briones-Roblero et al., 2017).

The gDNA was isolated using DNeasy^®^ Blood and Tissue kit (QIAGEN; Austin, TX, United States) according to the protocol of the manufacturer. The whole-genome sequencing was performed on the HiSeq2000 Illumina platform by Otogenetics Corporation (Norcross, GA, United States). The paired-end data were quality checked using FastQC v.0.11.3 (Babraham Institute, Cambridge, United Kingdom). Low quality reads were filtered using Trimmomatic v.036 (Bolger et al., 2014), and the filtered reads of high quality were *de novo* assembled using Velvet v.1.2.10 (Zerbino and Birney, 2008). Protein-coding, rRNA-coding, and tRNA-coding genes were identified using Prokka v.2.8,^1^ the Kyoto Encyclopedia of Genes and Genomes (KEGG) Automatic Annotation Server (KAAS) (KEGG Automatic Annotation Server^2^). Circular genome view was generated by CGView.^3^

Carbohydrate-active enzymes (CAZymes) were predicted on the dbCAN2 meta server^4^ using HMMER, DIAMOND, and HotPep algorithms, with their default parameters (Zhang et al., 2018). Only the CAZymes annotated with a minimum of two tools were retained.

### Bioinformatics Analyses of Xylanase-Ferulic Acid Esterase Gene

One copy of the xylanase-ferulic acid esterase gene (GenBank accession number MW981258), named *r13 fae* in this study, was identified in the *Rahnella* sp. ChDrAdgB13 genome. The predicted protein R13 Fae and its physicochemical characteristics, including molecular mass and isoelectric point (pI) of the predicted protein, were determined using the ProtParam tool^5^ (Gasteiger et al., 2005) and the signal peptide was predicted in the SignalP 5.0 server^6^ (Petersen et al., 2011). The likely sub-cellular localization of R13 Fae was determined in the TargetP-2.0 server^7^ (Emanuelsson et al., 2000). The Prosite database was used to predict potential *N*- and *O*-glycosylation sites^8^ (Sigrist et al., 2013).

The crystal structure for uncultured bacterium ferulic acid esterase protein (PDB ID: 6rzn.1) (Holck et al., 2019) was downloaded from the RCSB (Bookhaven Protein Data Bank) web site,^9^ and it was used as a template for the secondary structure. Prediction of the secondary structure elements and sequence alignment was done using ESPript 3.0 (Robert and Gouet, 2014).

### Phylogenetic Analysis of the Xylanase-Ferulic Acid Esterase

Putative esterases and glycosyl hydrolases of *Rahnella* species and some members of the family Yersiniaceae downloaded from Genbank^10^ were aligned with R13 Fae using default parameters in Clustal X v.2.0 (Larkin et al., 2007). A phylogenetic inference analysis by maximum likelihood was performed with PhyML 3.0 in the ATGC Montpellier Bioinformatics platform.^11^ The best amino acid substitution model for this set of data was estimated with the SMS software (Lefort et al., 2017) in the same platform using the Akaike Information Criterion (AIC) and was the WAG + G model (-lnL -2764.75, gamma parameter 0.564). The robustness of the nodes was assessed after 1,000 pseudo replicates, and the esterase putative sequence of *Enterobacter* sp. R4-368 (R9VK11) was included as an outgroup.

### Structural Modeling of R13 Fae

The 3D structural model of R13 Fae protein was generated by homology modeling in the SWISS-MODEL server^12^ using as a template the crystalized structure obtained by the X-Ray of uncultured bacterium ferulic acid esterase (PDB ID: 6rzn.1A). The best model was selected and validated in the Structural Analysis and Verification Server^13^ using the Ramachandran plot and Errat (Colovos and Yeates, 1993). The 3D structure of the R13 Fae protein was analyzed using the PyMol™ software v.2.5.2 (Schrödinger, 2021).

Predicted interactions between R13 Fae and ferulic acid, α-naphthyl acetate, and arabinoxylan were evaluated using docking analyses. Ligands were obtained in Pubchem^14^ and were geometrically optimized with the Gaussian software v.5.0.9 (Frisch et al., 2009) using AM1 and DFT [B3LYP/6-31G (d, p)] levels. Output files were converted to .pdb files, and the ligand files were viewed using the AutoDockTools software (ADT v.4.2) (Morris et al., 2009). To perform docking analyses, the Kollman charges for all atoms were computed prior to the addition of polar hydrogens to the R13 Fae, and torsion angles in the small flexible ligands were included in the Monte Carlo algorithm. The protein exploration and definition of the binding site were prepared using a GRID-based procedure (Goodford, 1985). All docking simulations employed the hybrid Lamarckian Genetic Algorithm with an initial population of 100 randomly placed individuals and 1 × 10^7^ energy evaluations. The interactions between the ligands and the R13 Fae were visualized using the ADT software v.4.2, and the figures were created using the PyMol™ software v.2.5.2.

### Growth Conditions of Bacterial Strains

*Rahnella* sp. ChdrAdgB13 was grown on Congo red agar plates (0.37 g L^–1^ K_2_HPO_4_, 0.27 g L^–1^ MgSO_4_, 1.88 g L^–1^ beechwood xylan, 0.2 g l^–1^ Congo red, 5 g L^–1^ gelatin, 15 g L^–1^ agar; BD Difco Sparks, MD, United States) and incubated at 28°C for 24–48 h.

*Escherichia coli* strain DH5α and the plasmid pJET were used for DNA amplification. *E. coli* strain BL21 and expression vector pET-38b(+) (Fermentas, St Leon-Rot, Germany) were used for protein expression. *E. coli* strains were grown in agar plates and/or broth Luria-Bertani (LB, JT Baker, Phillipsburg, NJ, United States) containing ampicillin (100 μg ml^–1^) and/or kanamycin (30 μg ml^–1^) where appropriate, and then they were incubated at 37°C overnight unless otherwise stated.

### DNA Isolation and Plasmid Construction

Bacterial DNA was extracted from axenic colonies using DNeasy^®^ Blood and Tissue kit (QIAGEN, Austin, TX, United States). From the gDNA of strain ChdrAdgB13 was amplified the coding region of the *r13 fae* gene (≈1.1 kb) by Touchdown PCR in a T100™ thermocycler (BIO-RAD), using the following primer pair: forward, FX: 5′ GGATCCTCAAAGCCTGCCGCAGGACGCCGAC 3′, and reverse, RX: 5′ GAACGCGGCCGC**TTA**TTTAAATATCTTCTTC TGCAC 3′ with restriction sites *Bam*HI and *Not*I (underlined) and stop codon (bold) (New England Biolabs, Beverly, MA, United States). PCR amplification was performed in a 25 μl final reaction volume consisting of 1X PCR buffer, 2.5 mM MgSO_4_, 0.2 mM dNTPs, 0.33 μM of each primer, 1.5 U Platinum *Taq* DNA Polymerase High Fidelity (Invitrogen, Carlsbad, CA, United States) and 50 ng ml^–1^ of DNA sample. The PCR thermal cycle program consisted of an initial denaturalization step for 2 min at 94°C followed by 15 cycles of 30 s at 94°C and 2 min at 72°C, 15 cycles of 30 s at 94°C and 2 min at 70°C, and 35 cycles of 60 s at 94°C, 60 s at 68°C, and 60 s at 68°C, with a final extension at 68°C for 10 min. An amplicon of ≈1.1 kb was visualized on 1.0% agarose gels using 7X Gel Red (Biotium Inc., Hayward, CA, United States) as a fluorescence agent and compared with a 1 kb base pair (bp) DNA ladder (New England Biolabs, Beverly, MA, United States). The amplicon was purified with the QIAquick^®^ Gel extraction kit (Valencia, CA, United States), cloned into a pJET1.2/blunt vector (Fermentas, St Leon-Rot, Germany), and transformed into chemically competent *E. coli* DH5α cells, generating the pJET1.2/*r13 fae* construct, and it was purified and sequenced in Macrogen Inc. (Seoul, South Korea).

The pJET1.2/*r13 fae* construction was subjected to double digestion with *Bam*HI and *Not*I (New England Biolabs, Beverly, MA, United States) at 37°C for 2 h. The *r13 fae* gene was directionally subcloned into the *Bam*HI and *Not*I restriction sites of the pET38b(+) expression vector. Recombinant plasmids [pET38b(+)/*r13 fae*] were transferred to *E. coli* BL21 cells for protein expression assays.

### Expression and Purification of R13 Fae

The recombinant xylanase-ferulic acid esterase was expressed in *E. coli* BL21 cells harboring the pET38b(+)/*r13 fae* construction. *E. coli* cultures were grown overnight in LB medium/kanamycin (30 μg ml^–1^), diluted 100-fold in LB broth, and shake-incubated at 37°C, 180 rpm, at OD_600*nm*_ = 0.6 cell density. IPTG (Sigma-Aldrich, St. Louis, MO, United States) was added to reach a 1 mM final concentration, and cultures were further incubated overnight at 28°C, 80 rpm. Subsequently, cell cultures were harvested by centrifugation (9,000 × *g* at 4°C for 15 min). The supernatant was used as a source of crude xylanase-ferulic acid esterase to reveal xylanase and esterase activity and determine the protein profile was determined using 10% SDS–PAGE.

Protein analyses were carried out by 10% SDS-PAGE using a Mini PROTEAN II system (BioRad, Hercules, CA, United States), following the method described by Laemmli (1970). Proteins in the gel were visualized by Coomassie Brilliant Blue R-250 (Sigma-Aldrich, St. Louis, MO, United States). Protein molecular weight (MW) was estimated with reference to a broad range of molecular weight protein standards (BioRad, Hercules, CA, United States). Gels were recorded and analyzed using a UVP DigiDoc-It Imaging System (Analytik Jena, CA, United States). Protein concentration was determined as described by Lowry et al. (1951), using Bovine Serum Albumin Proteins (BSA, Sigma-Aldrich, St. Louis, MO, United States) as standard.

R13 Fae was purified by ion exchange chromatography from the supernatant [*E. coli* BL21/pET38b(+)/*r13 fae*] corresponding to crude extract of xylanase-ferulic acid esterase. This extract was loaded onto an ion exchange resin with a packed volume of 3.37 ml, previously equilibrated with buffer A (400 mM NaCl, 50 mM histidine buffer, pH 6.0). The purification was carried out manually at a constant flow rate, and 1.0 ml fractions were collected. Fractions with xylanolytic activity were pooled, dialyzed against 50 mM histidine buffer pH 6.0, and analyzed by 10% SDS-PAGE. The purified R13 Fae xylanase-ferulic acid esterase was stored at 4°C for further study.

### Enzyme Activity Assays

The xylanolytic activity of R13 Fae on beechwood xylan was determined from the amount of reducing sugars released during incubation at 25°C for 60 min. Reducing sugars were quantified using the dinitrosalicylic acid method (DNS), with xylose as standard (Miller, 1959). An enzyme preparation of 100 μl was added to 0.9 ml of 50 mM citrate buffer, pH 6.0, containing 1% (w/v) beechwood xylan (Sigma-Aldrich, St. Louis, MO, United States) as substrate. One unit (U) of enzymatic activity was defined as the amount of enzyme required to produce 1 μmol of xylose per minute under assay conditions. All tests were carried out in triplicate and average values were recorded.

The esterase activity of R13 Fae on α-naphthyl acetate was determined by 10% native-PAGE in a discontinuous system using 0.19 M tris-glycine buffer, pH 8.3, for 4 h at 40 mA. The enzymatic reaction used to reveal the esterase in the electrophoretic run was based on the histochemical technique using 100 ml of 0.1 M phosphate buffer, pH 6.7, 2 ml of 1% α-naphthyl acetate in acetone, and 20 mg Fast Blue RR Salt (Sigma-Aldrich, St. Louis, MO, United States). The solution was incubated in the gel at 25°C until the appearance of the band (Oliver et al., 1991).

The amylolytic activity of R13 Fae was determined on 1% starch agar plates (10 g L^–1^ starch, 23 g L^–1^ agar dissolved in deionized water), where 100 ml of xylanase-ferulic acid esterase was added to a 5 mm hole, previously made with a Pasteur pipette on starch agar plates. These were incubated at 25°C for 6 h and stained with Lugol’s solution to document the enzyme activity around the hole.

### Biochemical Characterization of R13 Fae

The pH effect on the enzymatic activity of purified R13 Fae enzyme was evaluated at different pH values, ranging from 3.5 to 10.5 using beechwood xylan as the substrate (1% w/v). The substrate was prepared in 50 mM of different buffers: citrate (pH 3.5–6.0), phosphate (pH 6.0–8.0), and glycine-NaOH (pH 8.5–10.5) that were incubated at 25°C for 1 h. The stability of R13 Fae was evaluated in the same pH range and temperature for 2 h, without substrate. Thereafter, the remaining xylanolytic activity was measured under standard conditions and compared to the untreated enzyme activity.

The effect of temperature on the enzymatic activity of purified R13 Fae was determined by conducting the assay at different temperatures ranging from 15 to 55°C using as substrate 1% (w/v) beechwood xylan (Sigma-Aldrich, St. Louis, MO, United States). The substrate was dissolved in a 50 mM citrate buffer at pH 6.0 for 30 min. To evaluate enzyme thermostability, the purified R13 Fae was incubated at different temperatures (25, 35, and 45°C) in a 50 mM citrate buffer, pH 6.0. For determining the half-life of the enzyme, aliquots of the sample were collected at different time intervals and residual enzymatic activity was measured under standard conditions.

The xylanolytic activity and substrate specificity of R13 Fae were determined under optimal assay conditions using 1% (w/v) from oat spelt xylan, beechwood xylan, and carboxymethyl cellulose (CMC, Sigma-Aldrich, St. Louis, MO, United States). For determining the kinetic parameters *K*_*m*_ and *V*_*max*_ from the initial reaction rates for R13 Fae were obtained under optimal conditions of the activity using beechwood xylan as substrate at a concentration ranging from 0.1 to 1.0 mg. The *K*_*m*_ and *V*_*max*_ values were obtained by means of the non-linear least squares regression method using the Michaelis-Menten kinetics.^15^

To determine the effect of metal ions and other compounds on the enzymatic activity of R13 Fae, samples of the purified enzyme were incubated in 1.0% beechwood xylan (Sigma-Aldrich, St. Louis, MO, United States) dissolved in 50 mM citrate buffer at pH 6.0 and containing the following ions: Ca^2+^, Mg^2+^, Fe^2+^, Cu^2+^, Li^1+^, Na^1+^, Zn^2+^, Hg^2+^, or ethylenediaminetetraacetic acid (EDTA) and 2-mercaptoethanol (2-β-Me) at a final concentration of 1 and 5 mM for each one. Reaction mixtures were incubated with the different compounds at 25°C for 2 h. Xylanase activities were assayed under standard conditions and compared to a control without additions. Temperature and substrate data were compared by one-way analysis of variance (ANOVA) using Tukey’s test in the SigmaStat 3.5 software (Systat Software Inc., San Jose, CA, United States).

Zymogram analysis was conducted as previously described by Cano-Ramírez et al. (2017). Briefly, protein samples were separated in a 10% polyacrylamide gel co-polymerized with 2% beechwood xylan, under denaturing conditions. Protein samples were resuspended in SDS sample buffer with 2-β-Me, and the samples were boiled in a water bath for 5 min. After electrophoresis, the enzymes were refolded with 2.5% Triton-X100 in 50 mM citrate buffer, pH 6.0 at 25°C for 1 h. Thereafter, the gel was further incubated in the above-mentioned buffer at 40°C for 3 h at 30 rpm. Protein bands were visualized by Coomassie Brilliant Blue R-250 staining. Then, gels were washed two times with 0.1 M Tris-HCl buffer pH 8.0 at room temperature for 10 min at 30 rpm. Xylanase activity was visualized by staining the gel with Congo red (1 mg ml^–1^) for 15 min while being gently agitated, and then distained in 1 M NaCl for 10 min.

The hydrolysis products released by the action of recombinant xylanase-ferulic acid esterase on beechwood xylan were analyzed by thin layer chromatography (TLC). The reaction mixture consisted of adding 1 ml of purified R13 Fae enzyme to 500 μl of 2% beechwood xylan in 50 mM citrate buffer at pH 6.0. The reaction mixture was incubated at 25°C, and aliquot samples were taken to different time spans of between 0 and 120 h. A total of 30 μl of each sample and 1 μl of the xylan-oligomer standard (xylose, xylobiose, xylotetraose, and xylohexose) were spotted on a silica gel (Megazyme, Bray, Ireland). The hydrolysis products were separated using a solvent mix of butanol/ethanol/water (5:3:2 v/v/v). The plate was sprayed with sulfuric acid (15% v/v) and heated in a dry oven at 80°C for 15 min to visualize the xylan oligomers.

## Results

### Genome Analysis and CAZymes Enzymes

**FIGURE 1:**
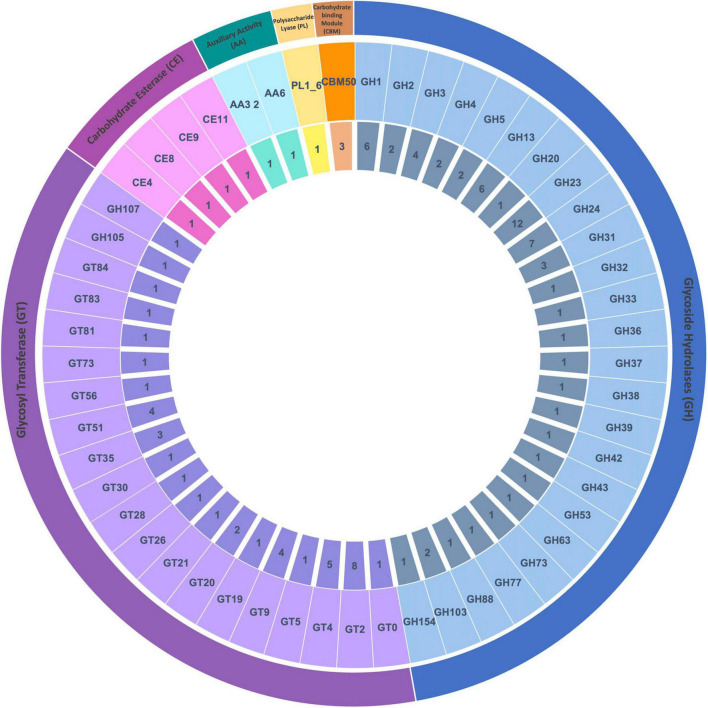
Distribution of CAZyme families from *Rahnella* sp. ChdrAdgB13. Genomic sequences predicted: 25 glycoside hydrolases (GH), 20 glycosyl transferases (GT), four carbohydrate esterases (CE), two auxiliary activities (AA), one polysaccharide lyases (PL), and one carbohydrate-binding module 50 (CBM50 associated with proteins LysM). From inner to outer rings are the numbers of genes and CAZyme families and classes, respectively.

The genome size of *Rahnella* sp. ChDrAdgB13 (JAEMGT000000000) was 5.73 Mbp, with 5,410 coding sequences. The genome analysis showed the highest number of genes related to carbohydrates metabolism (669), and in this subsystem, the following were found: 201 genes from monosaccharides metabolism were found, 154 from central carbohydrate, 71 from fermentation, and 109 from Di- and oligosaccharides (Table 1). Among completely annotated CAZymes, highlight 25 glycoside hydrolases (GH), 20 glycosyl transferases (GT), four carbohydrate esterases (CE), two auxiliary activities (AA), one polysaccharide lyase (PL), and one carbohydrate-binding module (CBM) families (Supplementary Table 1 and Figure 1). GH Families associated with cellulose, starch, and xylan hydrolysis and found in this study were 1, 3, 5, 13, 31, and 43 (Table 2).

**TABLE 1:** KEEG carbohydrates metabolic pathway genes identified *Rahnella* sp. ChdrAdgB13 genome.

Carbohydrate’s subsystem	Counts
Central carbohydrate metabolism	154
Aminosugars	17
Di- and oligosaccharides	109
One-carbon Metabolism	31
Organic acids	38
Fermentation	71
Sugar alcohols	34
Carbohydrates-no subcategory	1
Polysaccharides	13
Monosaccharides	201

**TABLE 2:** Identification of xylan, cellulose, and starch hydrolytic enzymes in the genome of *Rahnella* sp. ChDrAdgB13.

Hydrolysis	Genes	Enzyme	Family
Cellulose	1	Cellulase glycosyl hydrolase	GH5
	1	β-glucosidases (EC 3.2.1.21)	GH1, GH3
Starch	1	Cytoplasmic α-amylase (EC 3.2.1.1)	GH13
	1	α-glucosidases (EC 3.2.1.20)	GH31
	2	β-glucosidases (EC 3.2.1.21)	GH1, GH3
	1	Periplasmic β-glucosidase BgIX (EC 3.2.1.21)	GH3
Xylan	1	*endo*-1,4-β-D-xylanase (EC 3.2.1.8)	GH13
	2	α-xylosidases (EC 3.2.1.177)	GH31
	1	β-xylosidase (EC 3.2.1.37), partial	ND
	1	α-L-arabinofuranosidase	GH43
	1	Tannase and feruloyl esterase	ND

### Bioinformatics Analyses of Xylanase-Ferulic Acid Esterase Gene

**FIGURE 2:**
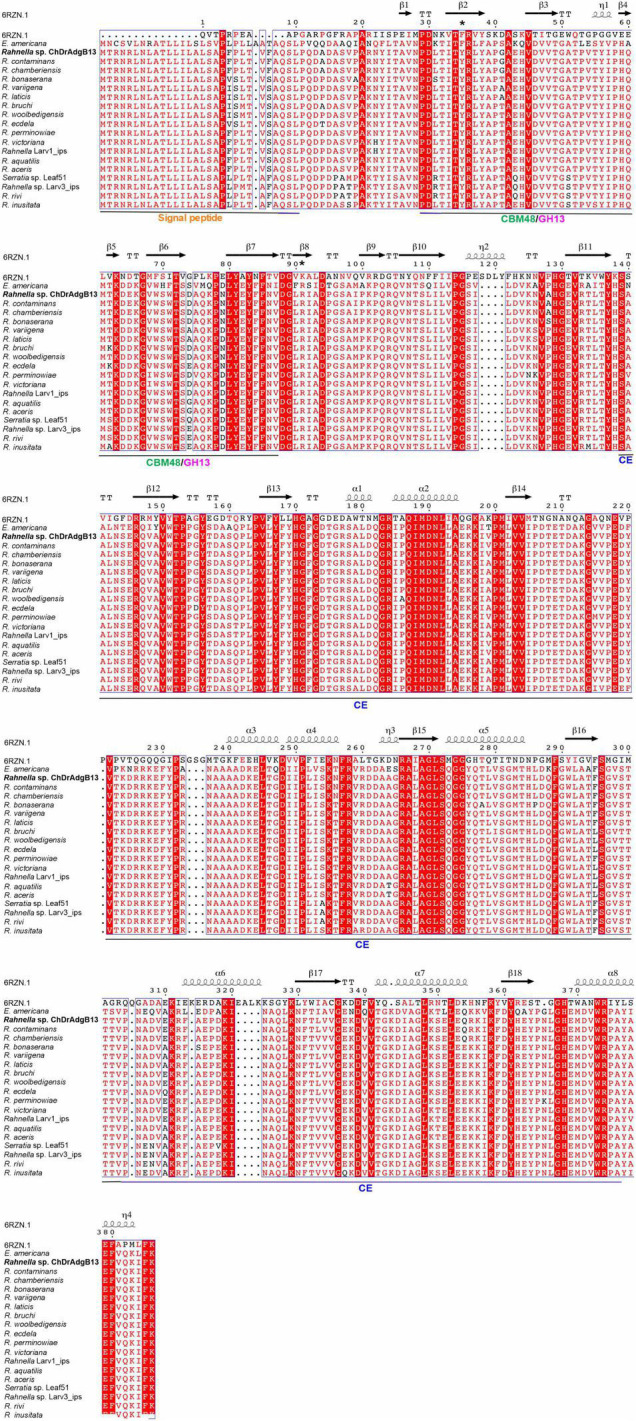
Multiple sequence alignment and secondary structure element assignment. The alignment included crystal structure from ferulic acid esterase 6RZN (A0A5S8WFA0_9BACT), xylanase-ferulic acid esterase gene (MW981258), esterase family protein of *Rahnella contaminans* Lac-M11 (A0A6M2B7F1), *R. aceris* (JAADJV00000000), *R. aquatilis* (H8NZD0), *R. bonaserana* (WP_217174935), *R. bruchi* (WP_120508294), *R. ecdela* (WP_217149683), *R. inusitata* (WP_120078305), *R. laticis* (JADOBK000000000), *R. perminowiae* (WP_217221065), *R. rivi* (WP_217204531), *R. variigena* (WP_120163835), *R. victoriana* (WP_09592434545), *R. woolbedingensis* (WP_120131505), *Rahnella* sp. Larv1_ips (MEHU01000001), *Rahnella* sp. Larv3_ips (WP_122094532), and members of the Yersiniaceae family such as *Ewingella americana* (WP_140475874), *Serratia* sp. Leaf51 (A0A0Q4MY82), and *Rouxiella chamberiensis* (WP_152327471). The complete amino acid sequences contained a signal peptide of 26 amino acids at the N-terminal end, Carbohydrate Binding Module (CBM) 48 (positions 51-106 aa) and Carbohydrate Esterase (CE) (positions 154-383 aa) with respect to crystal structure from ferulic acid esterase 6RZN (A0A5S8WFA0_9BACT). The helices are marked as α or β, based on the automatic assignment according to the template of the ferulic acid esterase (A0A5S8WFA0_9BACT) protein structure in the program ESPript 3.0. *Two substitutions in the SBS within the CBM48 domain, one from tryptophan to tyrosine, and another from tryptophan to arginin.

The *r13 fae* gene from *Rahnella* sp. ChdrAdgB13 has an open reading frame (ORF) of 1,182 bp, encoding for a predicted protein of 393 amino acid residues. The molecular mass and theoretical pI predicted for the enzyme were around 43.5 kDa and 5.94, respectively. The predictive subcellular localization of R13 Fae suggests that it is a secreted protein. The protein does not show *N*- or *O*-glycosylation sites. Identity analysis of the amino acid sequence of R13 Fae reveals that this enzyme is 92.3–99.9% identical to putative esterase and glycosyl hydrolases from *Rahnella* species known, 99.7% to *Rouxiella chamberiensis* (WP_152327471), 75.82% *Ewingella americana* (WP_140475874), 67.85% to *Serratia* sp. Leaf51 (A0A0Q4MY82) (Supplementary Table 2). The full amino acid sequence contains a signal peptide of 26 amino acids at the N-terminal end, a Carbohydrate Binding Module 48 (CBM) (positions 51-106 aa), and a domain of carbohydrate esterase (CE) (positions 154-383 aa) with respect to the crystal structure from ferulic acid esterase (6RZN; A0A5S8WFA0_9B ACT). Based on the sequence ferulic acid esterase secondary, and 3D structure, 18 β sheets, and eight α-helices are recognized (Figure 2), R13 Fae CBM48 corresponded to the GH13 family.

### PhyloGenetic Analysis of the Xylanase-Ferulic Acid Esterase gene

Phylogenetic analysis of R13 Fae CBM48, putative orthologous of *Rahnella* species, and glycosyl hydrolases of some members of the family Yersiniaceae integrated three consistent clusters (bootstrap value > 76%) (Supplementary Figure 1). Two of these clusters were integrated xylanase-ferulic acid esterase of *Rahnella* spp., and the third integrated orthologous of *R*. *rivi, R*. *inusinata, Rahnella*. sp., *Serratia* sp., and *Ewingella americana*. R13 Fae was nearer to those from *R*. *contaminans* Lac-M11 (A0A6M2B7F1), *R. chamberiensis* (WP_152327471), and *R. perminowiae* (WP_217221065).

To confirm that R13 Fae CBM48 has not the starch-binding site (SBS), an ML-phylogenetic analysis as was described in the MM section was performed with esterase sequences CBM48 of different species with and without SBS. The phylogeny showed the integration of two groups well support (>60%), one with a starch-binding site and the other without it where was included R13 Fae (Supplementary Figure 2).

### Structural Modeling of R13 Fae

**FIGURE 3:**
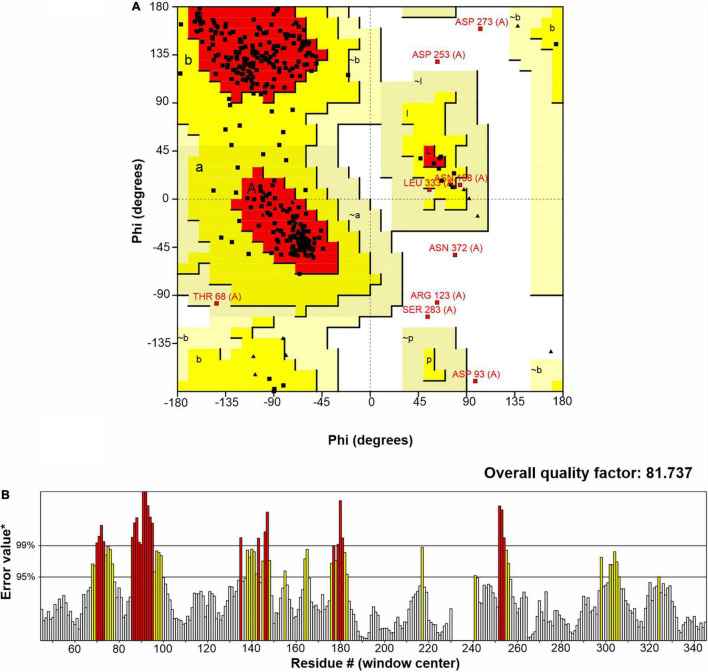
Structural model of the xylanase-ferulic acid esterase R13 Fae of *Rahnella* sp. ChDrAdgB13: **(A)** Ramachandran plot, residues in the most favored regions (A, B, L), residues in additional allowed regions (a, b, l, p), residues in generously allowed regions (∼a, ∼b, ∼l, ∼p), residues in disallowed regions (white region). **(B)** Errat plot and error-values*, based on the statistics of non-bonded atom-atom interactions in the structure.

R13 Fae shared an amino acid identity of 33.91% with the feruloyl esterase of uncultured bacterium (6RZN; A0A5S8WFA0_9BACT). The structural model selected for R13 Fae presented 71.39% of the amino acids with a score ≥ 0.2 in the 3D/1D profile and an average overall quality factor of 81.9172. The analysis of torsional angles Phi and Psi showed that 85.88% (260) of residues are in the most favored regions, 11.2% (34) of residues are in additional allowed regions, 1.0% (three) residues are in generously allowed regions, and 2.0% (six) of residues in the disallowed regions (Figure 3A). Also, the ERRAT test showed that the R13 Fae model was considered reliable with an overall quality factor of 81.73% (Figure 3B).

**FIGURE 4:**
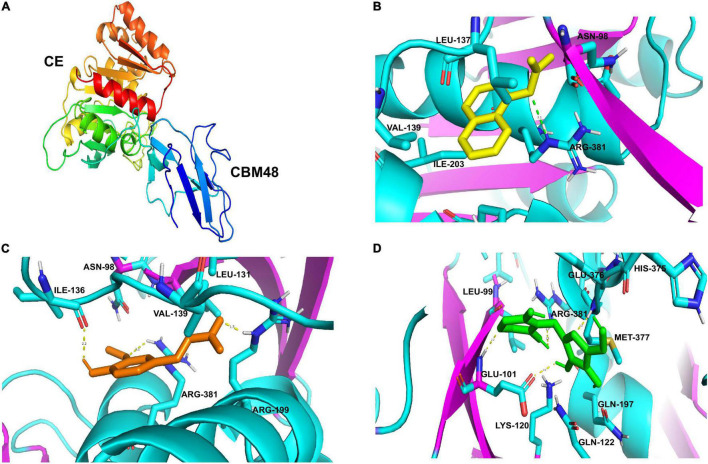
Predicted docked interactions of R13 Fae protein from *Rahnella* sp. **(A)** The cartoon structure: CE and CBM48 domains. The arrows indicate β-sheet, helical and α-helices structures. Cartoon diagrams highlight the interaction with ligands **(B)** α-naphthyl-acetate, **(C)** ferulic acid, and **(D)** arabinoxylan.

R13 Fae has a tertiary structure composed of two domains: The N-terminal CBM48 (residues 51-106) and the C-terminal CE domain (residues 154-383). The CBM48 displayed the typical fold of CBMs consisting of 7 β-strands, and the CE showed 7 β-strands and 8 α-helices (Figure 4A). Both domains have highly conserved residues that are involved in enzymatic catalysis, which included the catalytic triad (SER92, HIS375, ARG381).

Docking analyses have shown that R13 Fae has a higher affinity mainly with compounds having ester bonds such as α-naphthyl-acetate and ferulic acid, followed by the amylose from the starch, arabinoxylan, and lower affinity to amylopectin (Table 3). In the case of α-naphthyl-acetate, it presented binding energy and binding sites of −25.06 kJ mol^–1^ with ASN98, LEU137, VAL139, ARG199, ILE203, ASP378, and ARG381, respectively; with this last residue being part of a hydrogen bond with a distance of 3.1Å (Figure 4B). The binding energy with the ferulic acid was −24.51 kJ mol^–1^, the binding sites were ASN98, LEU131, ILE136, VAL139, ARG199, ILE203, ASP376, and ARG381, and the following residues were involved in the hydrogen bond: ARG199 (1.7Å), ILE136 (2.2Å), and ARG381 (3.1Å) (Figure 4C).

**TABLE 3:** Interaction, binding energy, and interaction residues of ligands with R13 Fae.

Substrate	α-nafthyl-acetate	Ferulic acid	Arabinoxylan	Amylopectin	Amylose

Interaction	Negative and frequent	Negative	Frequent	Negative	Frequent
Binding energy (kJ/mol^–1^)	ΔG = −25.06	ΔG = −24.51	ΔG = −17.65	ΔG = −2.59	ΔG = −23.59
Interaction residues	ASN 98 LEU 137 VAL 139 ARG 199 ILE 203 ASP 378 ARG 381	ASN 98 LEU 131 ILE 136 VAL 139 ARG 199 ILE 203 ASP 376 ARG 381	LEU 99 GLU 101 LYS 120 GLN 122 GLN 197 HIS 375 GLU 376 MET 377 ARG 381	PHE 187 GLU 243 PHE 244 TYR 245 ASN 248 ALA 250 ALA 251 ASP 253 PRO 315	LYS 355 GLU 359 ILE 363 LYS 364 PHE 365 TYR 367 HIS 368 GLU 369

The enzyme R13 Fae can also interact with arabinoxylan, the molecular docking showed binding energy of −17.65 kJ mol^–1^, and the binding sites were with residues LEU99, GLU101, LYS120, GLN122, GLN197, HIS375, GLU376, MET377, and ARG381; presenting a hydrogen bond with GLU101 (2.1, 2.2Å), MET377 (2.2Å), and ARG381 (2.5Å) (Figure 4D). Interestingly, all interactions showed an affinity with the ARG381 residue, which belongs to the catalytic triad of this enzyme, through hydrogens bonds.

Docking analyses showed an interaction between R13 Fae and amylose with a binding energy of −23.59 kJ mol^–1^, but less with amylopectin having a binding energy of −2.59 kJ mol^–1^ (Table 3). The binding sites for amylose were with residues LYS355, GLU359, ILE363, LYS364, PHE365, TYR367, HIS368, and GLU369, while for interaction with amylopectin the binding sites were with residues PHE187, GLU243, PHE244, TYR245, ASN248, ALA250, ALA251, ASP253, and PRO315.

### Expression, Purification, and Biochemical Characterization of R13 Fae Xylanase-Ferulic Acid Esterase

**FIGURE 5:**
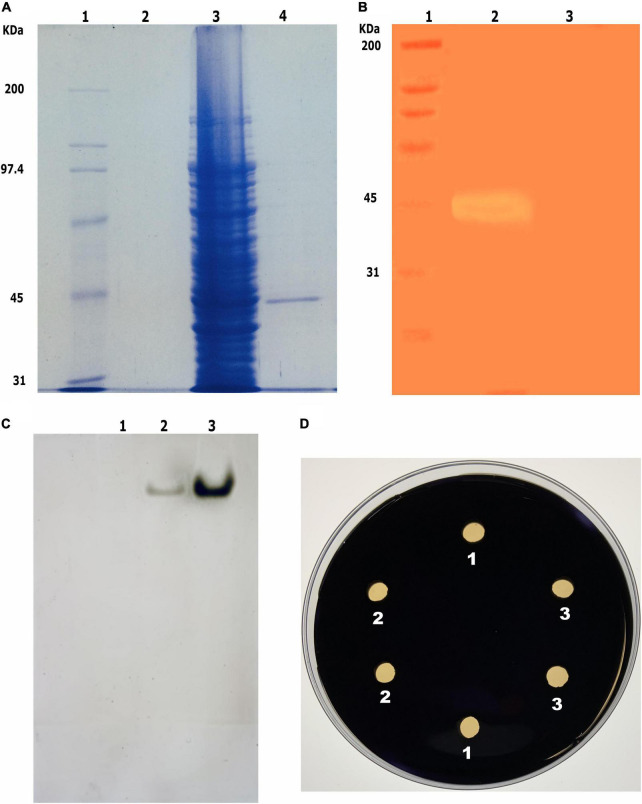
Purification, zymogram, and activity analysis of R13 Fae. **(A)** Purification of recombinant R13 Fae. Line 1, MW protein standard; line 2, non-induced soluble fraction medium; line 3, total protein extracts of transformed *E. coli* BL21/pET38b(+)/*r13 fae*; line 4, R13 Fae purified. **(B)** Zymogram of R13 Fae in 10% SDS-PAGE with 2% beechwood xylan. Line 1, MW protein standard; line 2, xylanolytic activity band of R13 Fae. **(C)** Activity of R13 Fae on α-naphthyl acetate in 10% native-PAGE. Line 1, non-induced soluble fraction medium; line 2, R13 Fae dialyzed; line 3, R13 Fae purified. **(D)** Activity of R13 Fae on starch on 1% (w/v) starch agar plates during 6 h at 25°C. We tested two clones of *E. coli* BL21/pET38b(+)/*r13 fae*. Line 1, non-induced soluble fraction medium; line 2, R13 Fae dialyzed; line 3, R13 Fae purified.

The profile of total protein R13 Fae in the soluble fraction of the bacterial lysate revealed the overexpression of polypeptide with an estimated molecular weight of 42.5 kDa corresponding to the expected molecular weight of R13 Fae xylanase. In comparison to non-induced cells, the heterologous protein was expressed at high levels by cells after induction. The recombinant protein was purified from the culture media and purified by ion exchange chromatography. SDS–PAGE analysis of extracts from the bacteria harboring the construct pET-38b(+)/*r13 fae* revealed a single protein band of R13 Fae of approximately of 42.5 kDa (Figure 5A). The zymogram analyses of purified R13 Fae performed under denaturing conditions, showed a single band that confirmed the xylanolytic activity and monomeric nature of the enzyme (Figure 5B). Also, R13 Fae showed affinity toward α-naphthyl acetate (Figure 5C), but no amylolytic activity was observed (Figure 5D).

**FIGURE 6:**
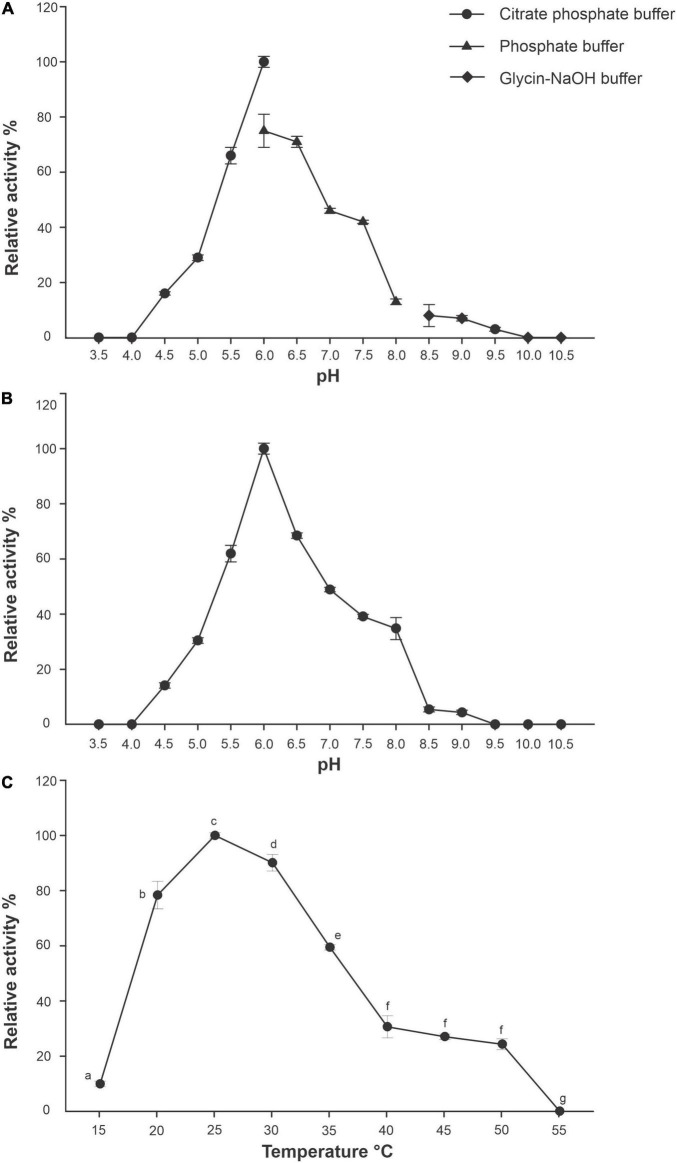
Effect of pH and temperature of R13 Fae activity and stability. **(A)** pH effect with different buffers (*n* = 3): citrate (pH 3.5–6.0), phoshate (pH 6.0–8.0), and glycine-NaOH (pH 8.5–10.5) incubated at 25°C for 60 min. **(B)** pH stability (*n* = 3), the protein was incubated in buffers mentioned above at different pH values, ranging from 3.5 to 10.5 at 25°C for 2 h. **(C)** Temperature effect (*n* = 3), the enzyme was incubated with 1.0% (w/v) beechwood xylan dissolved in 50 mM citrate buffer at pH 6.0 for 30 min at different temperatures (15–55°C). Different lower case letters indicate statistical significance (*p* < 0.001).

R13 Fae xylanase displayed optimal activity at pH 6.0 in the citrate buffer. The enzyme exhibited from 16 to 66% and from 3 to 71% of its maximal activity at different pH values ranging from 4.5 to 5.5, and from 6.5 to 9.5, respectively. The enzyme at pH 3.5, 10.0, and 10.5 was inactive (Figure 6A). According to pH stability assays, R13 Fae was highly stable across a pH range of 4.5–9.0, retaining between 66 and 71% of its original activity after 2 h of incubation at 25°C (Figure 6B).

The optimal temperature for R13 Fae was 25°C (Figure 6C); yet the enzyme showed from 10 to 90% of its original activity at a wide range of temperatures, between 15 and 50°C (Figure 6C) which were statistically significant (*F* = 815.39, *df* = 26, *p* < 0.001). The enzyme showed a half-life at pH 6.0 of 23 and 16 days at 25 and 35°C, respectively, but at 45°C the enzyme was stable for only 3 h.

**FIGURE 7:**
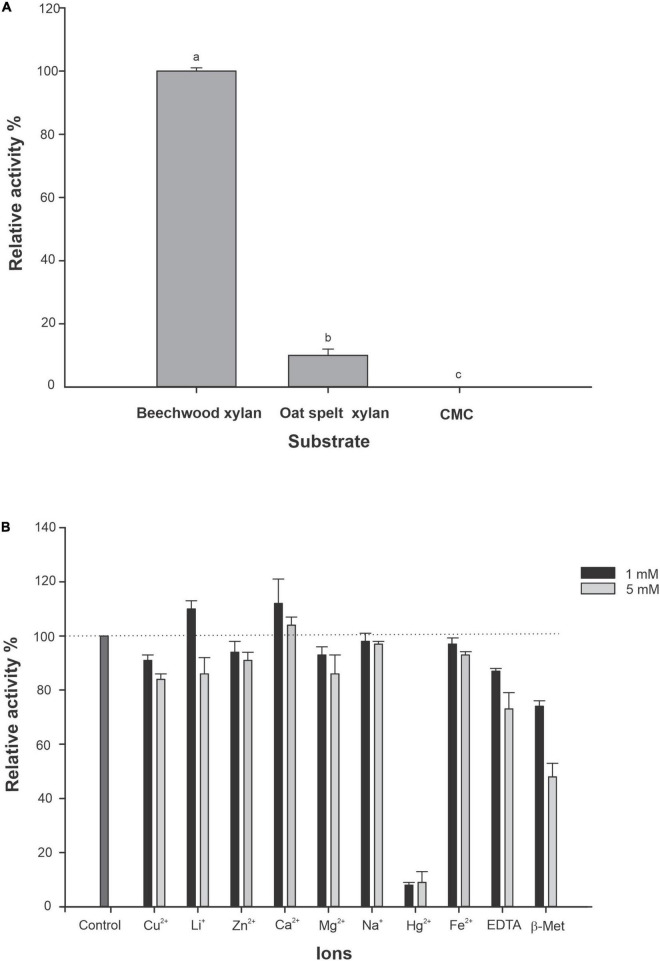
Substrate affinity of R13 Fae and effect of metal ions, EDTA and 2-β-Met on enzyme activity (*n* = 3). **(A)** The activity of R13 Fae was assayed using beechwood xylan, oat spelt xylan, and CMC at a final concentration of 1.0% (w/v) each in 50 mM citrate buffer pH 6.0 at 25°C. Different lower case letters indicate statistical significance (*p* < 0.001). **(B)** Effect of metal ions, EDTA and β-Met on R13 Fae activity at two concentrations 1 and 5 mM. Enzyme was incubated in 1.0% (w/v) beechwood xylan in 50 mM citrate buffer pH 6.0 containing 1 and 5 mM of Cu^2+^, Li^+^, Zn^2+^, Ca^2+^, Mg^2+^, Na^+^, Hg^2+^, Fe^2+^, EDTA, and 2-β-Met at 25°C for 2 h.

R13 Fae showed a higher affinity toward beechwood xylan (1%) and oat spelt xylan (1%), while no xylanolytic activity was detected on CMC (1%) (Figure 7A). These different affinities were statistically significant (*F* = 846.33, *df* = 8, *p* < 0.001). The *K*_*m*_ and *V*_*max*_ were 14 mg ml^–1^ and 0.598 μmol min^–1^ mg^–1^ of protein both at pH 6.0 and 25°C, using beechwood xylan in concentrations ranging from 0.1 to 1.0 mg ml^–1^.

The effect of metal ions, EDTA, and 2-β-Me both at 1 and 5 mM on the activity of R13 Fae is presented in Figure 7B. R13 Fae activity was enhanced by 10% with respect to control (enzyme without ions) with Ca^2+^ and Li^+^ (1 mM), whereas the presence of Cu^2+^, Na^+^, Mg^2+^, Fe^2+^, Zn^2+^, EDTA, and 2-β-Me decreased the activity between 10 and 40%, mainly at 5 mM concentration. The activity of R13 Fae was almost completely inhibited in the presence of Hg^2+^ (1 and 5 mM) (Figure 7B).

**FIGURE 8:**
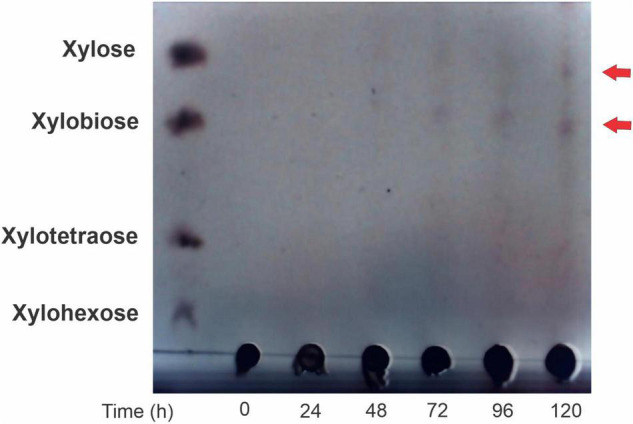
Thin layer chromatography of R13 Fae activity through cinetic time. Enzyme was incubated in 2% (w/v) beechwood xylan in 50 mM buffer citrates pH 6.0 at 25°C during 0–120 h.

The hydrolysis products of beechwood xylan yielded by the action of R13 Fae were xylobiose and xylose after 48 h of incubation time at 25°C and pH 6.0 (Figure 8).

## Discussion

Our findings showed that the genome size of *Rahnella* sp. ChDrAdgB13 is within the range of genomes sizes available for two isolates and 13 species of the genus *Rahnella* (4.91–5.78 Mbp) (Fabryová et al., 2018; Lee et al., 2020; Jeon et al., 2021; Brady et al., 2022). Among GHs found in this study that might have an ecological role relevant to the nutrition of these bark beetles, families 1, 3, 13, 31, and 43, which have been associated with the hydrolysis of structural and non-structural polysaccharides have specifically been highlighted. *In silico* analysis also showed the presence of genes coding for several accessory enzymes involved in xylan depolymerization, such as *endo*-1,4-α-D-xylanase (92.3–99.9%), α-xylosidases (91.08–98.67%), α-L-arabinofuranosidase (77.84–99.07%), tannase and feruloyl esterase (70.13–99.06), and β-xylosidase (53.03–96.27%) in the genome of *Rahnella* species and other species of the Yersinaceae family, that are highly conserved according to their amino acid identity (Supplementary Table 2). Among these accessory proteins, the *endo*-1,4-β-D-xylanase are fundamental enzymes, given that they hydrolyze 1,4-β-D linkages between the xylose residues integrating the homopolymeric backbone chain of xylan. Yet, the molecular characterization of the *endo*-1,4-β-D-xylanase from *Rahnella* sp. ChDrAdgB13 indicates that this enzyme is not typical xylanase, but a xylanase-ferulic acid esterase (R13 Fae) with two appended domains, CBM48 and CE, and without SBS.

This xylanase-ferulic acid esterase is present in all *Rahnella* species and some members of the Yersinaceae family (Figure 2 and Supplementary Table 2); however, their functional activity is yet to be demonstrated. Recently, it was reported that two strains of *Rahnella* sp. (Larv1 and Larv3) isolated from the bark beetle *I*. *typographus* have xylanolytic activity on plates (Fabryová et al., 2018), which could be explained by the presence of the GH YieL enzyme, an orthologous protein of R13 Fae, having amino acid identity between 92.36 and 94.91% (Figure 2).

This study has demonstrated that R13 Fae is accessory xylanase with capacities to hydrolyze xylan and ester compounds. Their inclusion in the phylogeny within sequence groups of species with the CBM48 domain without starch-binding linkage, suggests that this enzyme acts preferentially on ester-bonds of hemicellulose, whose main component is xylan. In addition, hydrolysis products, xylose, and xylobiose, identified in activity assays indicate that it acts on the ends of the xylan (Figure 8). The identification of xylobiose and xylose suggests that this enzyme has *exo*-activity, as has been previously observed with the thermostable xylanase of *Thermoanarobacterium* species (Zarafeta et al., 2020). These substrates derived from xylan hydrolysis are compounds of easy assimilation and can be used by other symbionts present in the intestine and by insects.

As suggested by activity assays with ester-bonded compounds, R13 Fae could also act on the ferulic acid of xylan. In fact, docking analyses showed similar binding energies between R13 Fae with α-naphthyl acetate and ferulic acid, which are more negative than with arabinoxylan. CBM48 and CE domains of R13 Fae act in consort, however, CE is the domain that involved most amino acids in the molecular interaction with different substrates (Figure 4 and Table 3). Thus, residues GLU101, MET377, and ARG381 interact with arabinoxylan; ILE136, ARG199, and ARG381 with ferulic acid; and only ARG381 with α-naphthyl acetate. Apparently, the ARG381 residue plays an important role in the interaction with these substrates and could be involved in the stability and thermostability of these interactions (McRee, 2012).

The CBM48 domain has been associated with the ability to hydrolyze starch (Janeèek et al., 2019); however, our activity assay on starch indicates that R13 Fae has no activity on this substrate. Docking analyses showed low binding energy between R13 Fae and amylopectin (ΔG = −2.59 kJ mol^–1^) but high binding energy with amylose (ΔG = −23.59 kJ mol^–1^) (Table 3), which is explained by the structural nature (branched or linear) of these compounds. Other studies with xylanase-ferulic acid esterase appended with this domain have also reported negative results with respect to starch hydrolysis (Peng et al., 2014; Holck et al., 2019). The inability of R13 Fae to degrade starch is associated with two substitutions in the SBS within the CBM48 domain, one from tryptophan to tyrosine, and another from tryptophan to arginine, as has been previously reported in other species unable to degrade starch (Machovič and Janeèek, 2008; Peng et al., 2014; Holck et al., 2019). These substitutions are also present in orthologous sequences of this enzyme in all *Rahnella* species.

Biochemical assays have shown that the recombinant R13 Fae from *Rahnella* sp. ChDrAdgB13 has characteristics favoring its activity within the insect’s gut (Figure 6A). The pH recorded (4.5–9.0) in which R13 Fae has activity agrees with the recorded gut pH (6.0–11.8) in some herbivorous insects (Sheng et al., 2014; Mason, 2020). The range also agrees with that reported for xylanases (pH 2.0–11) deposited in the “Braunschweig enzyme Database” (BRENDA^16^). This explains why R13 Fae is inactive at pHs between 3.5–4.0 and 10.0–10.5, which may be due to the protein denaturation or changing its tertiary structure. The possible molecular mechanism that explains this loss of activity is associated with the alteration in the surface load of the protein, which eliminates the electrostatic interactions stabilizing the tertiary structure (Zhou and Pang, 2018).

The optimal temperature of around 25°C, in which the R13 Fae enzyme demonstrates xylanolytic activity suggests that it is a mesophilic enzyme. This result agrees with that reported for uncovered metagenomic xylanases isolated from other environmental samples, such as soil, sediment, water, and effluent, from normal as well as extreme habitats (Verma and Satyanarayana, 2020). Despite this, most studies have documented that the xylanases of bacterial or fungal origin are thermophilic [see Chakdar et al. (2016) and citations therein], which might be indicative of the metabolic adaptation of this enzyme from *Rahnella* sp. ChDrAdgB13 to the gut conditions of bark beetle.

Thermostability results at 25 and 35°C, with a half-life of 23 and 16 days, respectively, indicate that the stability of R13 Fae decreased as the temperature increased to 45°C. This result confirms that it is an enzyme with mesophilic characteristics. The mesophilic nature and the thermostability of R13 Fae might be associated with the fact that bark beetles spend a large part of their life cycle under the pine bark feeding on phloem (Wood, 1982), where the inner and external bark temperatures are more stable and crucial to the metabolic activities of bark beetles (Becerril-Cruz pers. comm.).

Given that R13 Fae is capable of hydrolyzing xylan from Beech and to a lesser extent xylan from oats (10%), and no carboxymethylcellulose (CMC), apparently the enzyme does not present active sites for CMC (Kamble and Jadhav, 2012). In addition, the high *K*_*m*_ value (14 mg ml^–1^) indicates that it has a low affinity to hydrolyze Beech xylan, despite being able to hydrolyze this substrate under denaturing conditions. Similar values of *K*_*m*_ have been reported for xylanase Xyl1 produced by the fungus *Penicillium chrysogenum* P33 (*K*_*m*_ of 9.6 mg ml^–1^) using beechwood xylan as substrate (Yang et al., 2019) and in *Streptomyces* sp. S27 where the *K*_*m*_ value was 12.38 mg ml^–1^ (de Queiroz Brito Cunha et al., 2018).

Our findings with metal ions and chemicals compounds revealed that the xylanolytic activity of R13 Fae at concentrations of 1 and 5 mM is affected by ions Cu^2+^, Na^+^, Mg^2+^, Fe^2+^(from 2 to 26%) and Zn^2+^, EDTA, and β-mercaptoethanol (from 3 to 52%). This corroborates the results of previous studies performed with these enzymes, which reported that in presence of some of these ions the xylanases activity from *Sporisorium reilianum* was strongly inhibited (Pérez-Rodríguez et al., 2020). Despite this slight effect, our results suggest that R13 Fae maintained its functional and structural stability even in the presence of these ions. Other studies have also documented cases in which xylanases have increased their activity in the presence of Mg^2+^, Na^+^, Fe^2+^, and Zn^2+^ such as fungus *Lichtheimia ramosa* H71D where the xylanase activity is increased to 100% (Alvarez-Zúñiga et al., 2017).

It is known that these ions block the thiol groups of the protein located in its active sites, which are essential to maintaining its tertiary structure (Haq et al., 2012). In addition, enzymatic inhibition can also be produced by metallic ions and/or chemical agents associated with several factors, such as the presence of at least one sulfhydryl group at the active site, mainly in the amino acid cysteine, the oxidation due to cations that destabilize the conformational folding of the enzyme or well, leading to the formation of disulfide bridges in an irregular position of the protein (Ajsuvakova et al., 2020).

A notable result is that xylanolytic activity in R13 Fae increases 10 and 12% in the presence of Li^+^ and Ca^2+^ ions, respectively. The increase in xylanolytic activity by these ions has also been observed in other fungi, such as *L. ramosa* H71D where activity increased by 70% in the presence of Ca^2+^ and 21% in the presence of Li^+^ (Alvarez-Zúñiga et al., 2017). These ions are acquired by bark beetles through feeding; however, both must be regulated in the gut. Lithium ion (Li^+^) is toxic to cells in high concentrations and Ca^2+^ is a fundamental ion that participates in many physiological processes (Taylor, 1987).

In brief, this study evidences the ability of *Rahnella*, a gut-associated bacterium of *Dendroctonus* bark beetles, to hydrolyze xylan and its products are assimilated by its host and other gut microbes as a nutritional source. This ability adds to other known capabilities of this bacterium, such as its ability to degrade ester and lipid compounds (Briones-Roblero et al., 2017), recycle uric acid (Morales-Jiménez et al., 2013), and tolerate and degrade monoterpenes (Boone et al., 2013; Xu et al., 2015, 2016). These results demonstrate the relevant functional role of *Rahnella* in bacterial-insect interaction contributing to their fitness, development, and survival. Future research directed toward a metatranscriptomic, metaproteomic, and metabolomic approach could be used to study the chemistry relationship between microbial symbionts (bacteria, yeast, and filamentous fungi, mainly) and the molecules that insect synthetizes that may be related to processes such as colonization, detoxification, pheromone production, and nutrition of the bark beetles.

## Data Availability Statement

The datasets presented in this study can be found in online repositories. The names of the repository/repositories and accession number(s) can be found in the article/Supplementary Material.

## Author Contributions

RMP-M and CC-R: heterologous expression, purification, and biochemical characterization of xylanase-ferulic acid esterase and genome analysis. MFL: docking and helped draft the manuscript. MEH-L, AL-L, and AS-H: support in biochemical characterization of xylanase-ferulic acid esterase and revised draft of the manuscript. RMP-M, CC-R, FO, and GZ: conceived the study and participated in its design and wrote the draft and final manuscript. All authors read and approved the final version of this manuscript.

## Conflict of Interest

The authors declare that the research was conducted in the absence of any commercial or financial relationships that could be construed as a potential conflict of interest.

## Publisher’s Note

All claims expressed in this article are solely those of the authors and do not necessarily represent those of their affiliated organizations, or those of the publisher, the editors and the reviewers. Any product that may be evaluated in this article, or claim that may be made by its manufacturer, is not guaranteed or endorsed by the publisher.

## Footnotes

1
http://exon.gatech.edu/genemark/genemarks2.cgi

2
http://www.genome.jp/kegg/

3
http://cgview.ca

4
http://bcb.unl.edu/dbCAN2/

5
https://web.expasy.org/protparam/

6
http://www.cbs.dtu.dk/services/SignalP/

7
http://www.cbs.dtu.dk/services/TargetP/

8
https://prosite.expasy.org

9
http://www.rcsb.org

10
https://www.ncbi.nlm.nih.gov/genbank/

11
http://www.atgc-montpellier.fr/phyml/

12
https://swissmodel.expasy.org/

13
https://saves.mbi.ucla.edu

14
https://pubchem.ncbi.nlm.nih.gov/

15
http://biomodel.uah.es/metab/enzimas/MM-regresion.htm

16
https://www.brenda-enzymes.org/
