# Supplementary material for: Rahnella sp., a Dominant Symbiont of the Core Gut Bacteriome of Dendroctonus Species, Has Metabolic Capacity to Degrade Xylan by Bifunctional Xylanase-Ferulic Acid Esterase
Source: Front Microbiol. 2022 May 31;13:911269. doi: 10.3389/fmicb.2022.911269 (PMC9195170; doi:10.3389/fmicb.2022.911269)
Supplement: Supplementary file 1 [file Data_Sheet_1.DOCX]

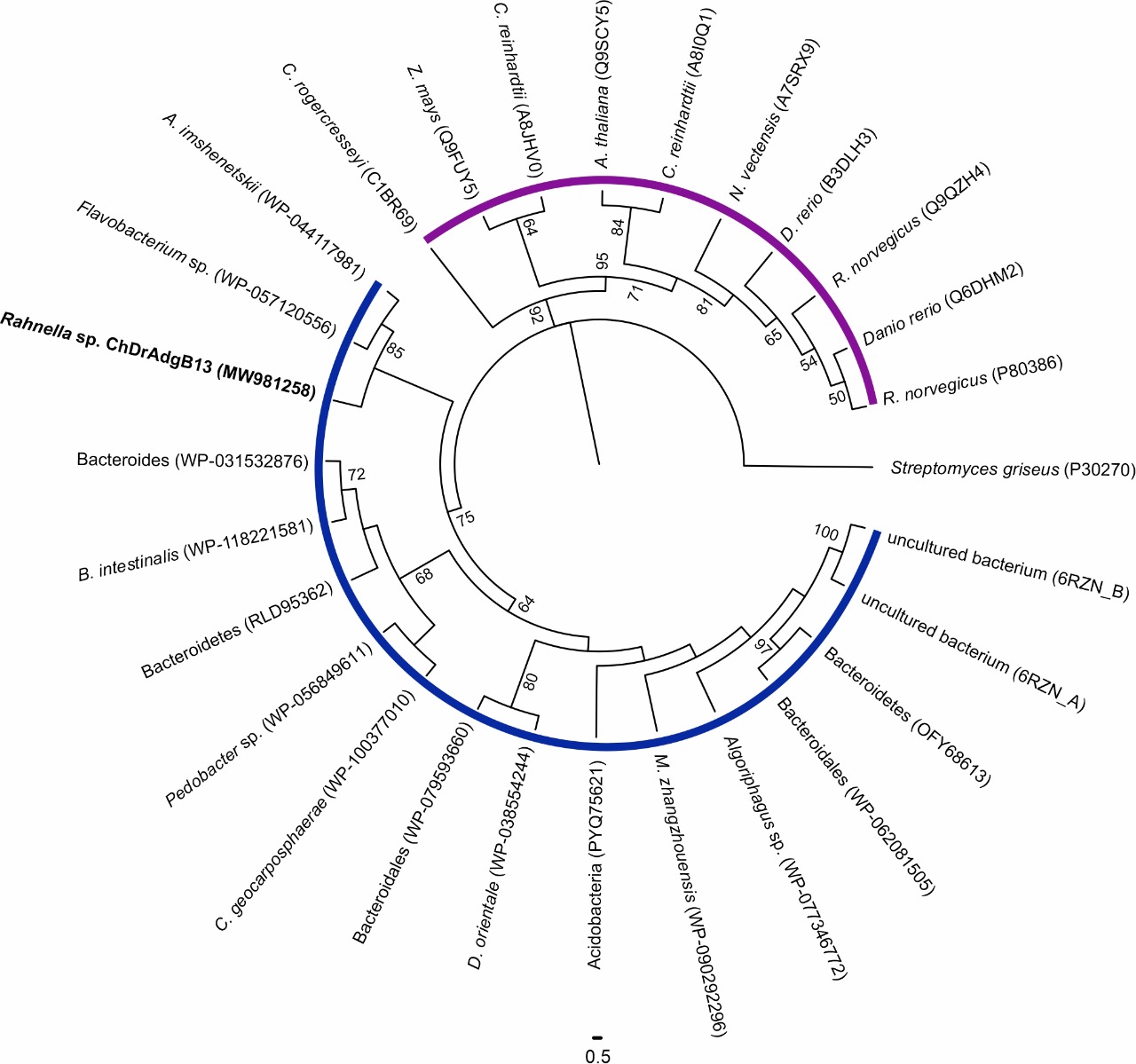


**FIGURE S2**. Maximum likelihood phylogenetic tree of starch binding (blue) and non-binding (purple) with CBM48 domains sequence and the representative sequences of GenBank and Uniprot databases. The VT+G model (-lnL 46167.73, gamma parameter 2.127) was used for the analysis. *Streptomyces griseus* (P30270) was used as an outgroup. The robustness at each node was assessed after 1000 pseudoreplicates and bootstrap support values are indicated for major nodes having 50% values. The scale bar indicates substitution/site. *A*. *imshenetskii* = *Alkaliflexus imshenetskii*; *B*. *intestinalis* = *Bacteroides* *intestinalis*; *C*. *geocarposphaerae* = *Chryseobacterium geocarposphaerae*; *D*. *orientale* = *Draconibacterium orientale*; *M*. *zhangzhouensis* = *Muricauda zhangzhouensis*; *R*. *norvegicus* = *Rattus norvegicus*; *D*. *rerio* = *Danio rerio*; *N*. *vectensis* = *Nematostella vectensis*; *C*. *reinhardtii* = *Chlamydomonas reinhardtii*; *A*. *thaliana* = *Arabidopsis thaliana*; *Z*. *mays* = *Zea mays*; *C*. *rogercresseyi* = *Caligus rogercresseyi*.
