# Supplementary material for: Rahnella sp., a Dominant Symbiont of the Core Gut Bacteriome of Dendroctonus Species, Has Metabolic Capacity to Degrade Xylan by Bifunctional Xylanase-Ferulic Acid Esterase
Source: Front Microbiol. 2022 May 31;13:911269. doi: 10.3389/fmicb.2022.911269 (PMC9195170; doi:10.3389/fmicb.2022.911269)
Supplement: Supplementary file 2 [file Data_Sheet_2.DOCX]

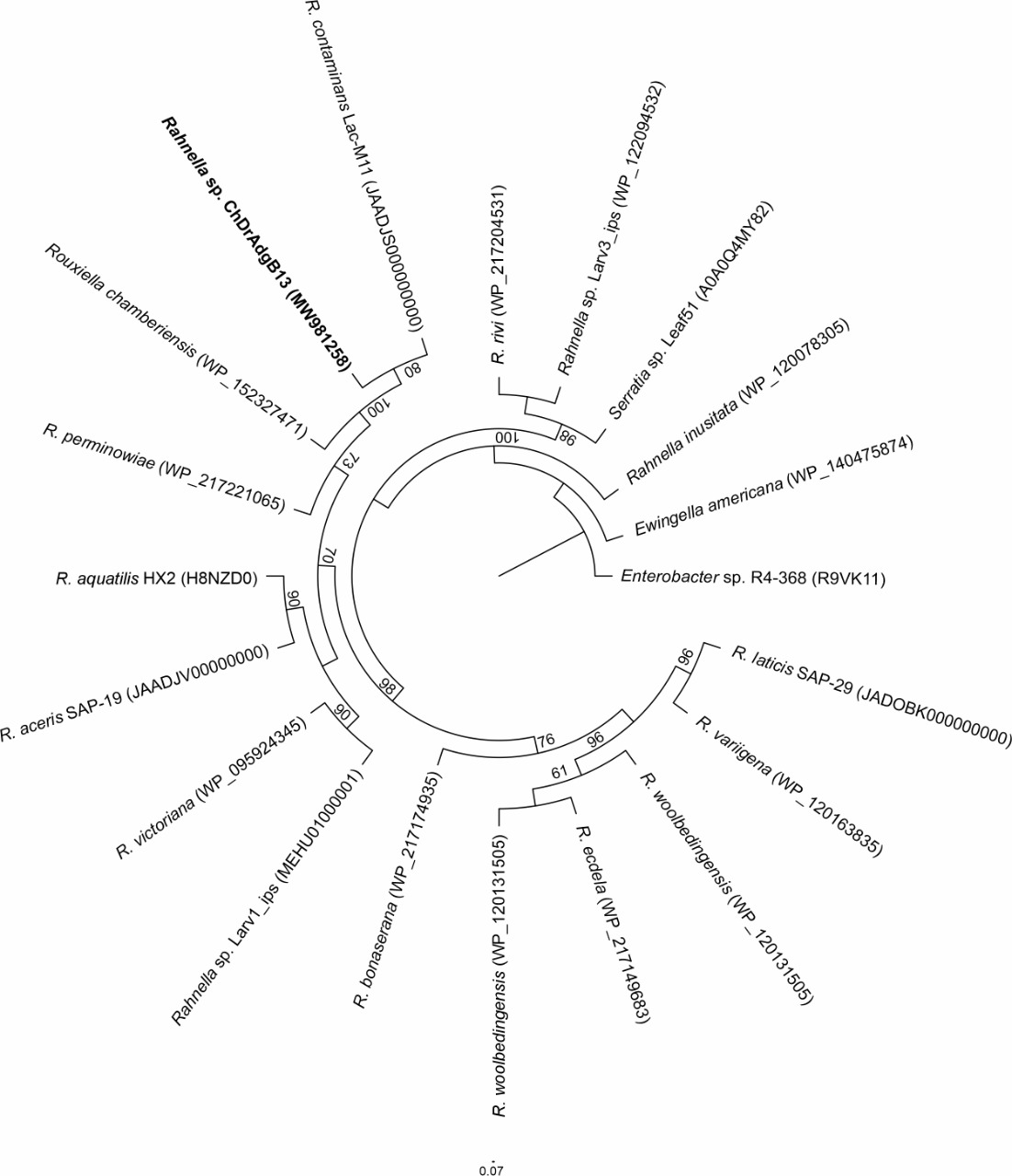


**FIGURE S1**. Maximum likelihood phylogenetic tree of xylanase-ferulic acid esterase sequence of *Rahnella* sp. ChDrAdgB13 and the representative sequences of GenBank and Uniprot databases. The WAG + G model (-lnL -2764.75, gamma parameter 0.564) was used for the analysis. Esterase putative sequence of the *Enterobacter* sp. R4-368 (R9VK11) was used as an outgroup. The robustness at each node was assessed after 1000 pseudoreplicates and bootstrap support values are indicated for major nodes having 50% values. The scale bar indicates substitution/site.
