# Supplementary material for: Rahnella sp., a Dominant Symbiont of the Core Gut Bacteriome of Dendroctonus Species, Has Metabolic Capacity to Degrade Xylan by Bifunctional Xylanase-Ferulic Acid Esterase
Source: Front Microbiol. 2022 May 31;13:911269. doi: 10.3389/fmicb.2022.911269 (PMC9195170; doi:10.3389/fmicb.2022.911269)
Supplement: Supplementary file 3 [file Table_1.DOCX]

**TABLE S1**. Identification of CAZy genes in *Rahnella* sp. ChdrAdgB13 genome.

| **Enzyme classes (GH)** | **Number of identified genes** | **Carbohydrate-active Enzymes** |
| --- | --- | --- |
| GH1 | 6 | β-glucosidase (EC 3.2.1.21); β-galactosidase (EC 3.2.1.23); and others |
| GH2 | 2 | β-galactosidase (EC 3.2.1.23); β-mannosidase (EC 3.2.1.25); and others |
| GH3 | 4 | β-glucosidase (EC 3.2.1.21); xylan 1,4-β-xylosidase (EC 3.2.1.37), and others |
| GH4 | 2 | α-glucuronidase (EC 3.2.1.139) enzyme involved in xylan degradation and others. |
| GH5 | 2 | Cellulase (EC 3.2.1.4); chitosanase (EC 3.2.1.132); β-mannosidase (EC 3.2.1.25); and others |
| GH13 | 6 | α-amylase (EC 3.2.1.1); pullulanase (EC 3.2.1.41); and others |
| GH20 | 1 | β-hexosaminidase (EC 3.2.1.52); lacto-N-biosidase (EC 3.2.1.140); and others |
| GH23 | 12 | Lysozyme type G (EC 3.2.1.17); peptidoglycan lyase (EC 4.2.2.-) and others |
| GH24 | 7 | Lysozyme (EC 3.2.1.17) |
| GH31 | 3 | Alpha amylase catalytic region (EC:3.2.1.20), α-glucosidase (EC 3.2.1.20); and others |
| GH32 | 1 | Invertase (EC 3.2.1.26); endo-inulinase (EC 3.2.1.7); and others |
| GH33 | 1 | Sialidase or neuraminidase (EC 3.2.1.18); trans-sialidase (EC 2.4.1.-) and others |
| GH36 | 1 | α-galactosidase (EC 3.2.1.22); and others |
| GH37 | 1 | α,α-trehalase (EC 3.2.1.28) |
| GH38 | 1 | α-mannosidase (EC 3.2.1.24); mannosyl-oligosaccharide α-1,3-1,6-mannosid as e (EC 3.2.1.114) and others |
| GH39 | 1 | α-L-iduronidase (EC 3.2.1.76); β-xylosidase (EC 3.2.1.37) |
| GH42 | 1 | β-galactosidase (EC 3.2.1.23) and others |
| GH43 | 1 | Arabinanase, α-L-arabinofuranosidase (EC 3.2.1.55); β-xylosidase (EC 3.2.1 0.37) and others |
| GH53 | 1 | Endo-β-1,4-galactanase (EC 3.2.1.89) |
| GH63 | 1 | α-1,3-glucosidase (EC 3.2.1.84); α-glucosidase (EC 3.2.1.20) and others |
| GH73 | 1 | Lysozyme (EC 3.2.1.17); mannosyl-glycoprotein endo-β-N-acetylglucosaminidase (EC 3.2.1.96) and others |
| GH77 | 1 | Amylomaltase or 4-α-glucanotransferase (EC 2.4.1.25). |
| GH88 | 1 | D-4,5-unsaturated β-glucuronyl hydrolase (EC 3.2.1.-). Unknown function |
| GH103 | 2 | Peptidoglycan lytic transglycosylase (EC 3.2.1.-) |
| GH154 | 1 | β-glucuronidase (3.2.1.31) |
