# Supplementary material for: Rahnella sp., a Dominant Symbiont of the Core Gut Bacteriome of Dendroctonus Species, Has Metabolic Capacity to Degrade Xylan by Bifunctional Xylanase-Ferulic Acid Esterase
Source: Front Microbiol. 2022 May 31;13:911269. doi: 10.3389/fmicb.2022.911269 (PMC9195170; doi:10.3389/fmicb.2022.911269)
Supplement: Supplementary file 4 [file Table_2.DOCX]

**TABLE S2**. Amino acid identities of the enzymes involved in xylan hydrolysis in *Rahnella* sp. ChDrAdgB13 and its orthologs in *Rahnella* species and members of the family Yersiniaceae.

|  | **% identity** | | | | |
| --- | --- | --- | --- | --- | --- |
| **Species** | **endo-xyl** | **α-xyl** | **β-xyl** | **α-L-ara** | **Tanna/ferul** |
| *Rahnella aceris* (GCA_011684115) | 95.41 | 92.19 | 77.27 | 93.20 | 80.84 |
| *Rahnella aquatilis* (CP003244.1) | 95.41 | 91.08 | 79.54 | 91.66 | - |
| *Rahnella bonaserana* (GCA_019049675) | 94.14 | 92.24 | 74.24 | 93.82 | 70.13 |
| *Rahnella bruchi* (GCA_003614975.1) | 95.41 | 91.75 | 73.48 | 94.76 | 79.51 |
| *Rahnella contaminans* (JAADJS000000000.1) | 99.9 | 98.67 | 96.21 | 99.07 | 99.06 |
| *Rahnella ecdela* (GCA_019049625) | 94.91 | 92.78 | 78.78 | 93.53 | 79.51 |
| *Rahnella inusitata* (GCA_003602055) | 92.62 | 92.63 | 53.03 | 88.34 | 80.63 |
| *Rahnella laticis* (JADOBI000000000.1) | 94.91 | 92.63 | 76.51 | 94.46 | 82.38 |
| *Rahnella perminowiae* (GCA_019049755) | 94.91 | - | 77.27 | 92.59 | 77.44 |
| *Rahnella rivi* (GCA_019049655) | 92.36 | 92.76 | 53.03 | 88.03- | 80.26- |
| *Rahnella variigena* (GCA_003602185) | 94.91 | 92.63 | 73.48 | 94.46 | 82.35 |
| *Rahnella victoriana* (GCA_004330295) | 94.91 | 93.07 | 77.27 | 92.28 | 81.54 |
| *Rahnella woolbedingensis* (GCA_003602095) | 95.67 | 92.78 | 77.27 | 94.15 | 80.07 |
| *Rahnella* sp. Larv1_ips (MEHU00000000) | 94.91 | 92.04 | 77.27 | - | 80.79 |
| *Rahnella* sp. Larv3_ips (MJIB01000000) | 92.36 | 92.78 | 53.78 | 88.0 | 80.26 |
| *Ewingella americana* (GCA_000735345.1) | 75.82 | - | - | - | - |
| *Serratia* sp. Leaf51 (LMLJ00000000) | 67.85 | 92.78 | 53.03 | 88 | 80.80 |
| *Rouxiella chamberiensis* (GCA_000951135.1) | 99.74 | - | - | 77.84 | - |

**endo-xyl =** endo-1,4-β-xylanase (EC 3.2.1.8); **α-xyl =** α-xylosidases (EC 3.2.1.177); **β-xyl =** β-xylosidase (EC 3.2.1.37)**; α-L-ara =** α-L-arabinofuranosidase**; Tanna/ferul =** Tannase and feruloyl esterase.
